# Incremental diagnostic value of coronary computed tomography angiography derived fractional flow reserve to detect ischemia

**DOI:** 10.1038/s41598-025-95597-4

**Published:** 2025-04-14

**Authors:** Isabelle Ried, Insa Krinke, Rafael Adolf, Markus Krönke, Seyed Mahdi Moosavi, Eva Hendrich, Albrecht Will, Keno Bressem, Martin Hadamitzky

**Affiliations:** School of Medicine and Health, Department of Cardiovascular Radiology and Nuclear Medicine, Technical University of Munich, TUM University Hospital, German Heart Center, Lazarettstrasse 36, 80636 Munich, Germany

**Keywords:** Coronary computed tomographic angiography, Fractional flow reserve, Myocardial ischemia, Coronary artery disease, Cardiology, Vascular diseases

## Abstract

Over the past decade, coronary computed tomographic angiography (CCTA) has been the most robust non-invasive method for evaluating significant coronary stenosis. Thanks to new technologies, it is now possible to determine the fractional flow reserve (FFR) non-invasively using computed tomographic (CT) images. The aim of this work was to evaluate the incremental diagnostic value of CT-derived FFR for ischemia detection. In this retrospective monocentric study, we investigated 421 patients who underwent CCTA and subsequent ischemia testing between 04/2009 and 06/2020. Endpoint was ischemia on a coronary vessel level assessed by CMR (*n* = 20), SPECT (*n* = 225), invasive angiography (stenosis ≥ 90%; *n* = 80) or invasive FFR (positive if ≤ 0.8; *n* = 96). CT-FFR was derived from CCTA images by a machine learning (ML) based software prototype. Patients averaged 66.5 [58.2–73.6] years of age and 72.7% (*n* = 306) were male. Overall, 52.5% (*n* = 221) had hypertension and 67.9% (*n* = 286) had hypercholesteremia. Logistic regression analysis on a per vessel base showed that the diagnostic model with CT-FFR plus CCTA had significantly better-fit criteria than the diagnostic model with CCTA alone (log-likelihood χ^2^ 230.21 vs. 192.17; p for difference < 0.001). In particular, the area under curve (AUC) by receiver operating characteristics curve (ROC) analysis for CT-FFR plus CCTA (0.87) demonstrated greater discrimination of hemodynamic ischemia compared to CCTA alone (0.83; p for difference < 0.0001). Combined CCTA and CT-FFR have improved diagnostic accuracy compared to CCTA alone in detecting ischemia on the coronary vessel level and thus could reduce the use of invasive coronary angiography in the future.

## Introduction

As coronary artery disease (CAD) remains one of the leading causes of death in many parts of the world, optimal diagnosis is consequently of great importance^1–3^. Current guidelines for the management of CAD in patients with intermediate pre-test probability (PTP) and suspected CAD initially recommend non-invasive tests such as stress testing, e.g. by cardiovascular magnetic resonance (CMR), myocardial perfusion single-photon emission computed tomography (MP-SPECT) or stress echocardiography, as well as computed tomographic coronary angiography (CCTA) if the patient has a low intermediate PTP^4,5^. In the European Society of Cardiology (ESC) guidelines for the treatment of chronic coronary syndromes published in 2019, the recommendation level of CCTA was even raised to class I (evidence level B)^5^. CCTA provides three-dimensional images of the coronary tree, which provide valuable information about the anatomy and possibly narrowing of the coronary arteries. In doing so, CCTA can accurately detect the presence of CAD and determine the extent and severity of stenotic lesions with high diagnostic performance^6^. Nevertheless, it showed low specificity for the identification of haemodynamically relevant coronary stenoses^7^ and thus provides only a limited guide for interventions^8^. Only 20–50% of patients who receive invasive coronary angiography (ICA) after CCTA have anatomically relevant CAD (> 50% stenosis) and even fewer have haemodynamic relevance^7,9,10^. For this reason, ICA may be appropriate after a non-invasive examination to decide on revascularisation options^5^ and, if necessary, to perform them directly^11^. Furthermore, to determine whether coronary stenosis is haemodynamically relevant, the fractional flow reserve performed during ICA (iFFR) is currently the diagnostic standard^5,12^. iFFR relates the pressure in the coronary artery distal to the stenosis to the myocardial blood flow during maximal arteriolar vasodilation and can therefore provide information on the extent to which a particular stenosis is haemodynamically relevant and whether revascularisation of the stenosis is warranted^12^. At FFR values ≤ 0.80, distal coronary pressure is 80% of aortic pressure at maximal vasodilation and thus a lesion is considered to cause ischaemia^13,14^. Significant advances in imaging techniques have been made in recent years, so that it is now possible to determine a computed tomography (CT) -derived FFR using CCTA images^6^. In the first CT-FFR versions, a combination of anatomical data with computational fluid dynamics algorithms enabled an assessment of blood flow in the coronary artery tree^6,15^. In newer versions, the CT-FFR is solely machine learning (ML)-based^16^. CT-FFR thus offers a non-invasive alternative to iFFR, reducing the risks associated with ICA for the patient^15^, although ICA is classified as a low-risk procedure and is performed regularly, complications can still arise^17^. As the diagnostic relevance of CT-FFR is not yet fully established due to its novelty, the aim of this study was to assess the additional diagnostic value for the detection of lesion-specific ischaemia.

## Materials and methods

### Study sample

**Fig. 1:**
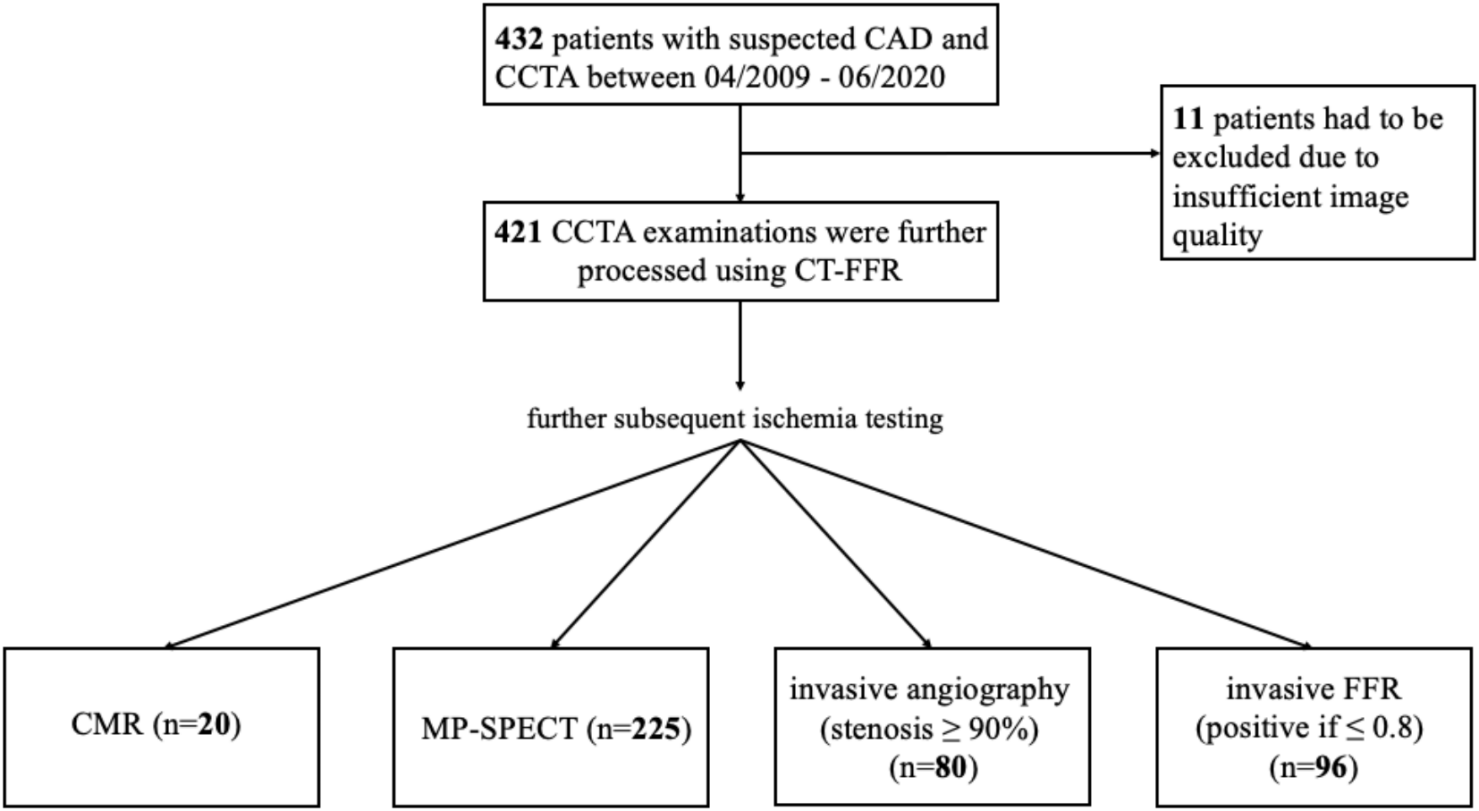
Study enrolment. *CCTA* coronary computed tomographic angiography; *CMR* cardiovascular magnetic resonance; *CT-FFR* computed-tomography derived fractional flow reserve, *MP-SPECT* Myocardial perfusion single-photon emission computed tomography.

This retrospective study included all consecutive 421 patients with suspected CAD who underwent CCTA and subsequent ischaemia testing within 90 days between April 2009 and June 2020. Ischaemia tests were either stress CMR (*n* = 20), SPECT (*n* = 225), invasive angiography (stenosis ≥ 90%; *n* = 80) or invasive FFR (positive if ≤ 0.8; *n* = 96) (Fig. 1). Patients with suspected CAD due to cardiac arrhythmia, stable chest pain, dyspnoea, risk profile and before or after pulmonary vein isolation were included.

Patients under 18 years, with congenital heart disease, an acute life-threatening condition or lack of sinus rhythm during the examination were excluded from this study. Written informed consent was obtained before the examination. A questionnaire collecting age, weight, height, family disposition, comorbidities and cardiac risk factors, symptoms and current medication was also completed before the examination. Regular use of antihypertensive medication or systolic blood pressure above 140 mmHg was considered as hypertension.

Diabetes mellitus was defined as a fasting blood glucose level above 7 mmol/L, pathologic oral glucose tolerance test or therapy with insulin or oral antidiabetic agents. Nicotine abuse was categorised as ‘never’, ‘former’ and ‘current smoker’. Former and current smokers were summarised under the criterion ‘nicotine’. Familial predisposition was defined as the presence of CAD in first-degree relatives younger than 65 years for women and younger than 55 years for men. Laboratory values collected included renal and thyroid values, as well as laboratory values for total cholesterol, low-density lipoprotein, high-density lipoprotein and triglycerides. The Morise risk score^18^ and the Framingham risk score^19^ were then determined from these cardiovascular risk factors.

The study was approved by the local ethics committee (Ethikkommission der Technischen Universität München, Munich, Germany). All CT scans were acquired in accordance with the relevant guidelines and regulations.

### Coronary CTA acquisition and analysis

Image acquisition was performed using a 192-slice dual-source CT scanner until 04/2014 (Somatom Flash) and a 384-slice dual-source CT scanner from 04/2014 to 06/2020 (Somatom Force), all Siemens Healthineers, Forchheim, (Germany).

Each of the 421 patients had their blood pressure and heart rate monitored before the examination. In the absence of contraindications, patients were administered intravenous β-blocker to achieve a heart rate of less than 60 beats per minute. In addition, sublingual administration of nitroglycerin was given to vasodilate the coronary arteries if systolic blood pressure was above 100 mmHg.

Coronary arteries were then analysed using the first 15 of the 18 segments according to the simplified classification of the American Heart Association classification^20^. The protocol for CT imaging has already been described in detail^21^. Each segment with a diameter > 1.5 mm was evaluated by two experienced radiologists, of whom at least one had read a minimum of 400 cardiac CT scans. In case of disagreement, these were resolved by consensus. The CCTA analyses were performed blind to the results of the ischaemia tests. The severity of the stenosis referred to diameter stenosis and categorically assessed as follows: no stenosis (0%), minimal obstruction (1-24%), mild obstruction (25-49%), moderate stenosis (50-69%), severe stenosis (70–89%) and high-grade stenosis (≥ 90%). Consequently, a stenosis was diagnosed from a lumen narrowing of ≥ 50%.

### CT-FFR computation

CCTA examinations were further investigated using ML-based CT-FFR (cFFR version 3.5.2 S Healthineers, Erlangen, Germany; not commercially available). For this purpose, coronary arteries with a minimum diameter of 1.5 mm were segmented using the simplified 15-segment model and ML-based CT-FFR was calculated. The ML application was trained using 12,000 synthetically generated 3D coronary models with varying anatomy and severity of CAD^16^. A reduced-order computational fluid dynamics (CFD) model was used to compute the pressure and flow distribution within the coronaries. Quantitative anatomical features along the coronary arteries and the FFR value calculated at this location were extracted. The ML-model was then trained to learn the relationship between the anatomical features and the FFR-value calculated with the CFD-model^16^. CT-FFR was measured distal to the stenosis, just behind the pressure drop caused by the stenosis. CT-FFR values ≤ 0.8, were considered haemodynamically significant^22^.

### CMR

In stress CMR, a stress perfusion defect was interpreted as ischaemia^23^. The CMR examinations were performed with a 1.5 Tesla machine (Siemens). Pharmacological stress was performed with a vasodilator (adenosine).

### MP-SPECT

In MP-SPECT, myocardial hypoperfusion in the sense of reduced retention of radionuclide tracers (technetium-99 m) during stress compared to radionuclide tracer retention at rest was interpreted as ischaemia^24^. Stress was induced either by physical exercise or by the administration of a vasodilator (adenosine or regadenoson at the discretion of the perfoming physician).

### ICA and iFFR

The iFFR measurement was performed according to standard practice^25^ in the cardiac catheterisation laboratory of the German Heart Centre Munich. iFFR values ≤ 0.8 were considered haemodynamically significant^13,14^.

### Endpoints and statistical analysis

The endpoint was defined as ischemia on a coronary vessel level, assessed either by noninvasive testing (MP-SPECT or perfusion stress CMR) or by invasive angiography, on which either a stenosis > 90% or an iFFR value of ≤ 0.8 were considered positive.

Categorical variables are presented as frequencies and percentages. Continuous variables are presented as means ± SDs or median [interquartile range] as appropriate. The diagnostic performance of CCTA and CT-FFR for detecting ischemia on a coronary vessel level and on a per patient level was reported as sensitivity, specificity, negative predictive value (NPV), positive predictive value (PPV) and accuracy with corresponding 95% confidence intervals (CIs). With respect to the endpoint, comparison between CT-FFR and CCTA se well as all incremental prognostic values of combine models were determined by area under the receiver operating characteristic curve (ROC) analysis^26^. The combined models were constructed according to the effect values in a multivariate logistic regression model. A two-sided p-value of < 0.05 was considered statistically significant. Statistical analyses were performed using R software (version 4.2.0; R Foundation).

## Results

### Patient characteristics

The study population consisted of 421 patients with CCTA examination between 04/2009 and 06/2020 and subsequent ischemia testing (Fig. 1). Mean age was 66.5 [58.2–73.6] years, 73% (*n* = 306) were male and a quarter had typical angina complaints prior the examination (*n* = 106, 25.2%). Significantly more men had ischaemia than women (*p* = 0.0048), patients with positive evidence of ischaemia also suffered significantly more frequently from an increased body mass index (*p* = 0.01), the other baseline characteristics showed no statistical differences (Table 1). A comparison between ischemia modalities showed that the CMR group was significantly younger, had more men and a higher BMI compared to the other groups. In addition, the FFR, CTA stenosis and SPECT groups had higher levels of hypercholesterolaemia and risk scores (Morise and Framingham risk scores), indicating a higher cardiovascular risk factor in these groups (Table 2).

**Table 1:** Baseline characteristics.

Parameter	All patients (*n* = 421)	Patients without ischemia (*n* = 308)	Patients with ischemia (*n* = 113)	*p*-value
Age (years)	65.6 ± 10.4	65.6 ± 10.3	65.7 ± 10.6	0.89
Male	306 (72.7)	213 (69.2)	93 (82.3)	**0.0068**
Diabetes	53 (28.1)^a^	34 (11)	19 (16.8)	0.14
BMI	26.5 ± 3.8 ^b^	21.1+-11.1	24.3 ± 11.4	**0.01**
Hypertension	221 (58.9)^c^	160 (51.9)	61 (54)	0.74
Hypercholesterolemia	286 (67.9)	208 (67.5)	78 (69)	0.81
Positive family history	148 (35.2)	107 (34.7)	41 (36.3)	0.82
Smoking	137 (36.9)^d^	99 (32.1)	38 (33.6)	0.81
NYHA III-IV	14 (3.9)^e^	10 (3.25)	4 (3.54)	>0.99
Morise risk score	11.9 ± 2.4 ^f^	11.6 ± 2.3	12.4 ± 2.61	**0.0028**
Framingham risk score	13.2 ± 9.7	12.3 ± 9.28	15.6 ± 10.3	**0.0039**
Symptoms				
Risk profile	101 (23.9)	82 (26.9)	19 (16.8)	0.1
Dyspnoea	38 (9.03)	25 (8.2)	13 (11.5)	0.58
Typical angina	106 (25.2)	79 (25.9)	27 (23.9)	0.92
Atypical chest pain	47 (11.2)	29 (9.51)	18 (15.9)	0.18
Arrhythmia	42 (9.9)	33 (10.8)	9 (7.96)	0.69
Positive ischemic testing	34 (8.1)	21 (6.89)	13 (11.5)	0.31
Post stenting	9 (2.1)	4 (1.31)	5 (4.42)	0.15
Pre surgery	4 (0.9)	2 (0.656)	2 (1.77)	0.58

Mean ± SD; Total No. of patients (%). *BMI* body-mass-index; *NYHA* New York Heart Association-Classification; Post-stening: CCTA after coronary stenting, pre surgery: CCTA to rule out obstructive CAD before Surgery or interventional valve replacement. ^a^Available in 377 patients; ^b^available in 346 patients; ^c^available in 375; ^d^available in 371; ^e^available in 358 patients; ^f^available in 418 patients. Significant values are in bold.

**Table 2:** Baseline characteristics patient comparison between the ischemia modalities.

Parameter	CMR (*n* = 20)	FFR (*n* = 96)	CTA-stenosis ≥ 90% (*n* = 80)	SPECT (*n* = 225)	*p*-value (ANOVA)
Age (years)	56.9 ± 12.9	65.1 ± 9.76	65.9 ± 11.4	66.5 ± 9.64	**0.001**
Male	17 (85)	62 (64.6)	66 (82.5)	161 (71.6)	**0.033**
Diabetes	2 (10)	9 (9.38)	15 (18.8)	27 (12)	0.28
BMI	25.5 ± 7.68	19.1 ± 12.2	24 ± 12.7	22.1 ± 10.3	**0.013**
Hypertension	9 (45)	43 (44.8)	43 (53.8)	126 (56)	0.27
Hypercholesterolemia	7 (35)	66 (68.8)	52 (65)	161 (71.6)	**0.0086**
Positive family history	10 (50)	38 (39.6)	30 (37.5)	70 (31.1)	0.21
Nicotine	7 (35)	34 (35.4)	29 (36.2)	67 (29.8)	0.64
NYHA III-IV	0 (0)	7 (7.29)	3 (3.75)	4 (1.78)	0.069
Morise Risk Score	10.3 ± 2.06	11.5 ± 2.34	12.6 ± 2.76	11.7 ± 2.25	**0.00026**
Framingham Risk Score	10.3 ± 5.88	11.1 ± 7.94	16.4 ± 10.6	13.2 ± 10	**0.0017**
Symptoms					
Risk profile	5 (25)	16 (16.8)	10 (12.5)	70 (31.4)	**0.0045**
Dyspnoea	0 (0)	11 (11.6)	8 (10)	19 (8.52)	0.58
Typical angina	7 (35)	32 (33.7)	19 (23.8)	48 (21.5)	0.18
Rule out	3 (15)	5 (5.26)	14 (17.5)	25 (11.2)	0.15
Arrhythmia	1 (5)	6 (6.32)	5 (6.25)	30 (13.5)	0.19
Positive ischemic testing	3 (15)	10 (10.5)	11 (13.8)	10 (4.48)	0.053
Post stenting	0 (0)	1 (1.05)	5 (6.25)	3 (1.35)	0.09
Preoperative	0 (0)	0 (0)	2 (2.5)	2 (0.897)	0.54

Mean ± SD; Total No. of patients (%). *BMI*: body-mass-index; nicotine: former and current smokers; *NYHA*: New York Heart Association-Classification; Rule out: in case of atypical angina pectoris symptoms; preoperative: before intervention or surgery. Significant values are in bold.

**Fig. 2:**
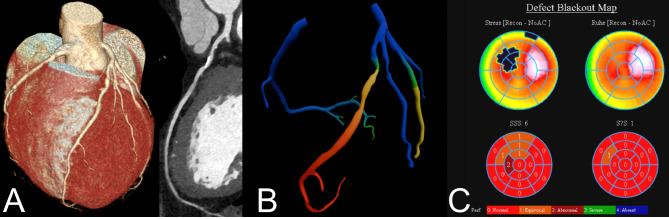
Representative image example. (**A**) CCTA shows a calcified 50–70% stenosis of the proximal LAD. (**B**) CTT-FFR with a pressure drop at stenosis level, the change from green to yellow identifies a FFR value of 0.8. (**C**) Myocardial perfusion scan shows a moderate stress perfusion defect in the LAD vessel territory.

A representative image example is shown in Fig. 2.

### Per-vessel diagnostic performance

Of the 938 vessels examined with CCTA, CT-FFR showed haemodynamically relevant lumen narrowing (CT-FFR ≤ 0.8) in 352 vessels (37.5%). No significant difference in diagnostic performance was observed between CT-FFR and CCTA with respect to the detection of ischemia at the coronary vessel level (AUC_CT−FFR_, 0.81 [95% CI, 0.77–0.85] vs. AUC_CCTA_, 0.83 [95% CI, 0.8–0.87], *p* = 0.249). Per-vessel diagnostic performance was similar between CT-FFR and CCTA (Table 3). The combination of CT-FFR and CCTA proved to be a better classifier for the primary endpoint than CCTA alone. (AUC_CT−FFR+CCTA_, 0.87 [95% CI, 0.83–0.9] vs. AUC_CCTA_, 0.83 [95% CI, 0.8–0.87], *p* < 0.001).

**Table 3:** Per-vessel diagnostic performance.

	CT-FFR	CCTA
Sensitivity	0.79 [0.59–0.99]	0.86 [0.64–1.06]
Specificity	0.69 [0.62–0.77]	0.68 [0.61–0.75]
PPV	0.30 [0.24–0.37]	0.31 [0.25–0.38]
NPV	0.95 [0.84–1.06]	0.97 [0.85–1.08]
Accuracy	0.71 [0.64–0.78]	0.71 [0.64–0.78]

Numbers in parentheses indicate 95% confidence interval. *CT-FFR* computed-tomography derived fractional flow reserve; *CCTA* coronary computed tomographic angiography.

**Fig. 3:**
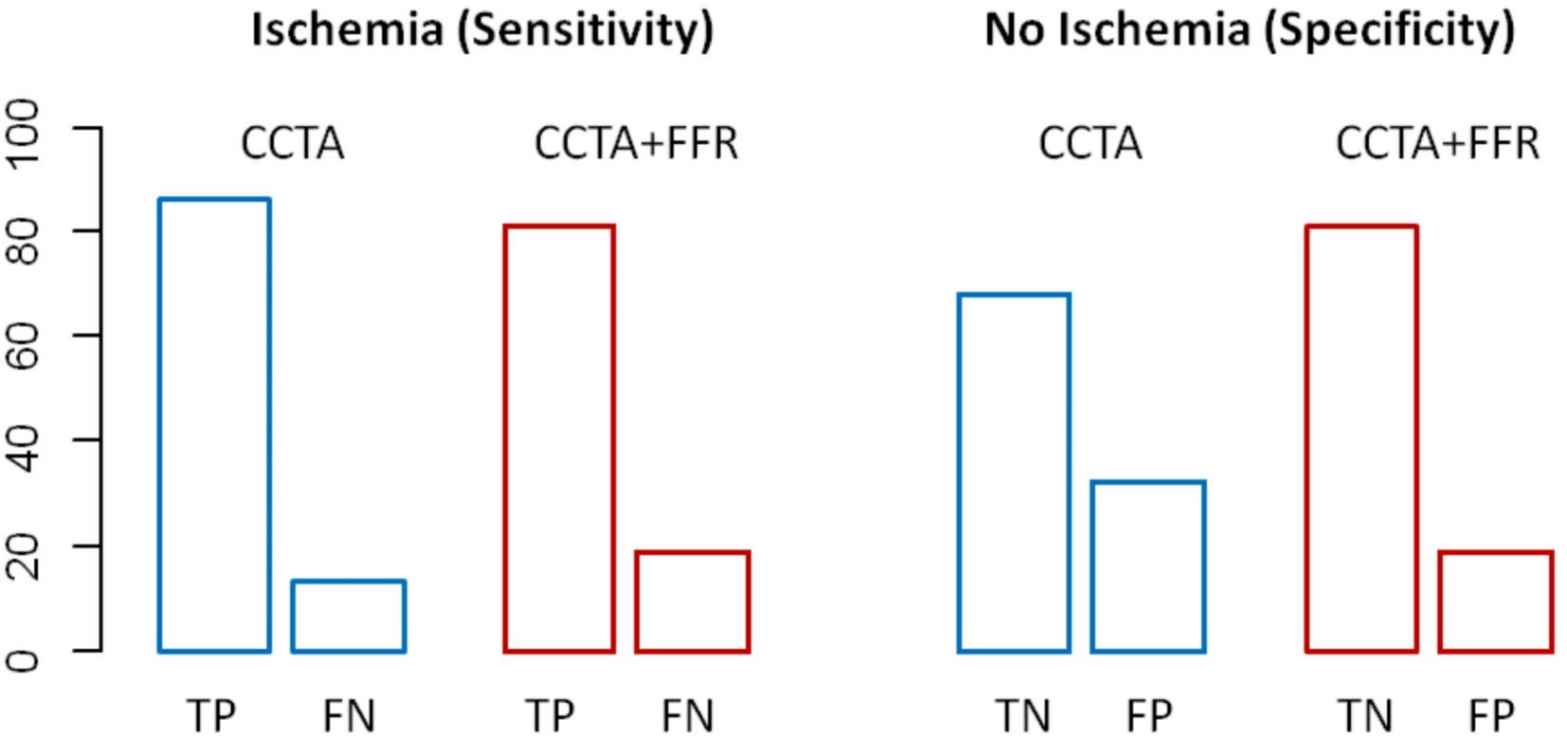
True and false positives and negatives of CCTA_alone_ and the combined model of CCTA + CT-FFR. *CCTA* coronary computed tomographic angiography; *CT-FFR* computed-tomography derived fractional flow reserve; *TP* true positive; *FP* false positive; *TN* true negative; *FN* false negative.

**Fig. 4:**
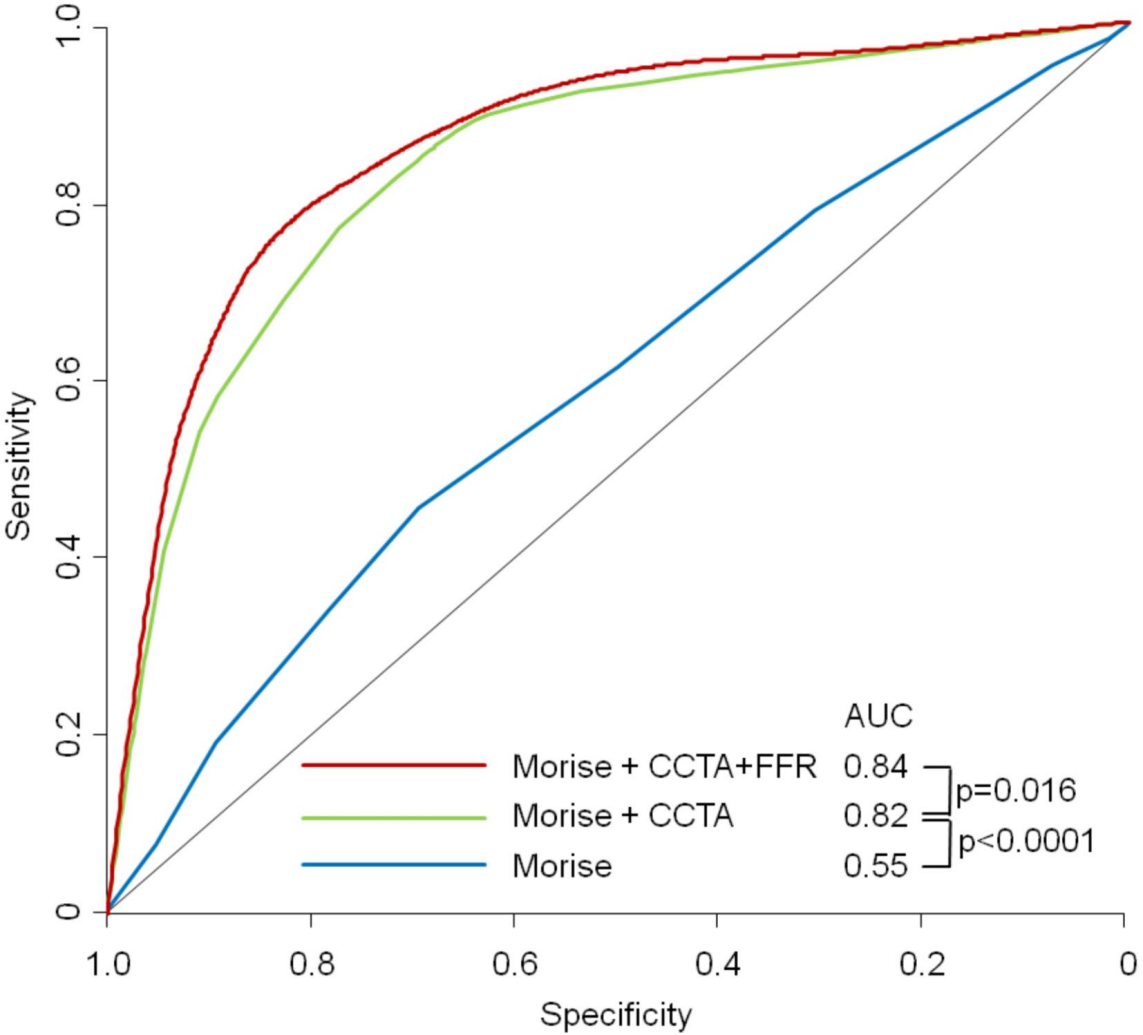
ROC-Curves for a stepwise model adding to Morise score firstCCTA and then CT-FFR for predicting ischemia on a per vessel-level. *AUC* area under the receiver-operating characteristic curve; *CCTA* coronary computed tomographic angiography; *CT-FFR* computed-tomography derived fractional flow reserve.

Furthermore, logistic regression analysis showed better fit criteria for the diagnostic model of CT-FFR plus CCTA than for the one with CCTA_alone_ (log->likelihood χ^2^ 230.21 vs. 192.17; *p* < 0.001). The analysis of the true and false positive CCTAs showed that the addition of CT-FFR can primarily identify false-positive CCTAs (CCTAs (high-grade stenoses without ischaemia) (Fig. 3). In a model adding stepwise CCTA and CT-FFR to Morise risk score. there was a significant improvement both for adding CCTA and CT-FFR from AUC = 0.545 to 0.815 to 0.839 (p for improvement < 0.0001 and 0.0161 resp.) (Fig. 4).

There was no performance difference of the fully combined model between the older and newer scanner generation (AUC 0.834 and 0.820 resp) but the incremental value of FFR-CT was not statistical significant with the older scanner generation (p for improvement 0.12 and 0.046 resp.)

### Per-patient diagnostic performance

**Fig. 5:**
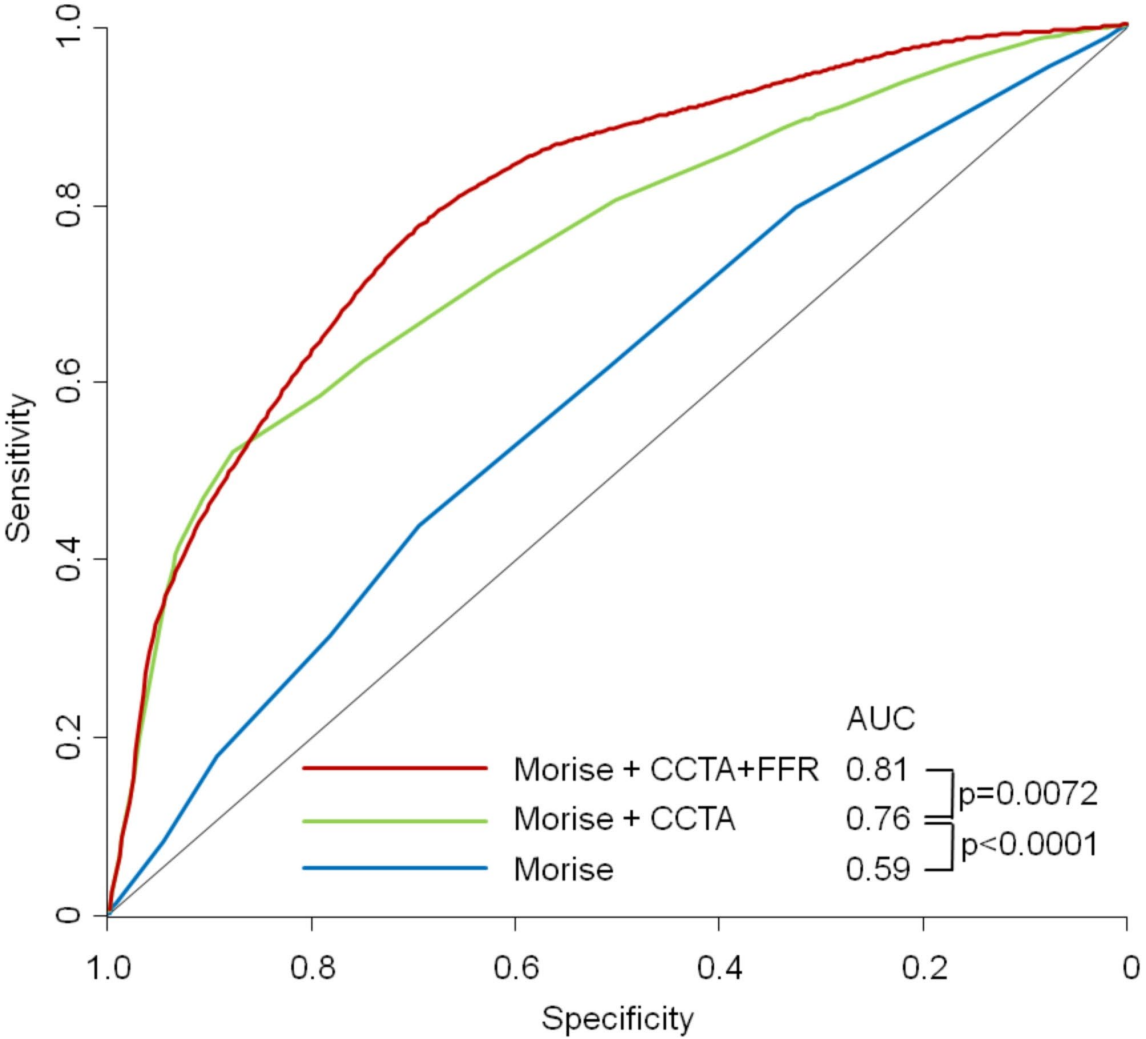
ROC-Curves for a stepwise model adding to Morise score firstCCTA and then CT-FFR for predicting ischemia on a per patient-level. *AUC* area under the receiver-operating characteristic curve; *CCTA*:coronary computed tomographic angiography; *CT-FFR* computed-tomography derived fractional flow reserve.

On a patient-level there were *n* = 18 positive results on SPECT, *n* = 15 on FFR, *n* = 77 on ICA and none on CMR. No significant difference in diagnostic performance was observed between CT-FFR and CCTA with respect to the detection of ischemia on a per-patient level (AUC_CT−FFR_, 0.76 [95% CI, 0.70–0.81] vs. AUC_CCTA_, 0.75 [95% CI, 0.70–0.80], *p* = 0.77). Per-patient diagnostic performance was also similar between CT-FFR and CCTA (Table 4). As in the per-vessel diagnostic, the multivariate analysis was supplemented by the clinical parameters (Morise Score). As in the per vessel analysis, adding stepwise CCTA and CT-FFR to Morise risk score. significant improved outcome prediction both by adding CCTA and CT-FFR (p for improvement < 0.0001 and 0.0072 resp.) (Fig. 5).

**Table 4:** Per-patient diagnostic performance.

	CT-FFR	CCTA
Sensitivity	0.89 [0.65–1.12]	0.93 [0.67–1.15]
Specificity	0.38 [0.30–0.46]	0.31 [0.24–0.39]
PPV	0.34 [0.27–0.42]	0.32 [0.25–0.39]
NPV	0.91 [0.68–1.12]	0.92 [0.66–1.16]
Accuracy	0.52 [0.43–0.60]	0.47 [0.39–0.55]

Numbers in parentheses indicate 95% confidence interval. *CT-FFR* computed-tomography derived fractional flow reserve; *CCTA* coronary computed tomographic angiography.

## Discussion

In this monocentric retrospective study, patients with evidence of obstructive CAD in CCTA and subsequent ischemia testing either by CMR, MP-SPECT, ICA or iFFR were investigated to what extent CT-FFR provides additional benefit in the diagnosis of ischaemia. Interestingly, the combination of CT-FFR and CCTA proved to be a more accurate classifier compared to using CCTA alone, supporting their complementary role in evaluating CAD.

Overall, with *n* = 421 patients, we analysed more patients than other comparable studies. Nevertheless, the baseline characteristics were very similar, and the data can thus be compared well. As in the literature, the average age in our study was in the mid-60s, more than 2/3 of the patients were male, and BMI tended to be slightly elevated. The frequency of typical risk factors such as dyslipidaemia, hypertension or diabetes were also comparable similar^27–30^.

Regarding our endpoint, previous studies showed significantly higher diagnostic accuracy with the use of CT-FFR^28,29,31–34^. The ROC curves in the literature demonstrated a higher diagnostic power of CT-FFR with AUC values of 0.81–0.9 compared to CCTA with AUC values of 0.66–0.75 ^27,30,31,35^. The DISCOVER-FLOW study, the DeFACTO study or the NXT study also demonstrated higher specificity, sensitivity and accuracy of CT-FFR compared to CCTA^27–29^. We did not find these differences, on the contrary, our analysis revealed similar to equal values for sensitivity (0.79 [0.59–0.99] vs. 0.86 [0.64–1.06]), specificity (0.69 [0.62–0.77] vs. 0.68 [0.61–0.75]) or accuracy (0.71 [0.64–0.78] vs. 0.71 [0.64–0.78]). This could be due to the multicentre study design of the above-mentioned studies and the high image quality in our study. However, it should be noted that the evaluation of data from different centres can also lead to distortions, in contrast to the evaluation of data from one centre, where the evaluation is always the same. Nevertheless, we found significantly higher diagnostic performance with the combination of CT-FFR and CCTA compared to CCTA alone (AUC 0,87 vs. 0.83; *p* < 0.001). When analysing the true and false-positive and -negative results of CCTA and the combined model of CCTA + CT-FFR, we were able to show impressively that the additional value of CT-FFR lies mainly in the identification of false-positive CTAs (high-grade stenoses without ischaemia). The addition of CT-FFR thus considerably increases the diagnostic accuracy of the non-invasive method. By adding clinical parameters (Morise risk score) to the multivariate model, a better predictive value could be achieved. Multimodal approaches that combine clinical and imaging procedures lead to significantly higher AUC values and thus to improved diagnostics^35^. As the combination of CCTA + CT-FFR has not been investigated in other studies, the values are only comparable to a limited extent. Nevertheless, they impressively demonstrate the significantly higher diagnostic accuracy of the additional use of CT-FFR in identifying haemodynamically relevant coronary stenoses.

In contrast to the ML-based CT-FFR used in this study, some of the earlier studies relied on a CFD-based CT-FFR, which limits comparability. However, Coenen et al. demonstrated convincingly that employing an ML approach, integrating pattern recognition and computerized learning, significantly enhanced the positive predictive value of CCTA for assessing the hemodynamic severity of coronary stenosis at both the vessel and patient levels. This substantial improvement was observed when compared to the utilization of a prior hybrid computational fluid dynamics approach^36^.

Since Choi et al. have shown that incomplete functional revascularisation is an independent predictor of major adverse cardiac events (MACE), effective stenosis elimination should be the goal of intervention^37^. A prerequisite for this is reliable diagnostics, which is why it is particularly interesting that the SYNTAX III trial was able to show that treatment recommendations based on CCTA and CT-FFR were highly consistent with those of invasive investigations^38^. In a recently published retrospective study of a single center, Becker et al. were able to show that, with the same outcome or number of MACE after one year, a decrease in invasive procedures was associated with an additional CT-FFR to CCTA^39^. As a result, the added benefit of CT-FFR demonstrated here and in the literature could help to adequately detect ischemia in patients and avoid further costly invasive diagnostic procedures^7,28,40^. In addition to reducing diagnostic costs, a reduction in the number of patients who experience complications during or after ICA contributes to a reduction in the costs to the health system caused by these complications. Reducing the burden on the health system and its resources is a strong argument for incorporating CT-FFR into regular medical practice.

Given that our CCTA examinations were conducted with state-of-the-art equipment and followed the recommended imaging protocol of the Society of Cardiovascular Computed Tomography, it’s reasonable to conclude that our CT-FFR results are unequivocally reliable.These recommendations included, for example, the administration of beta-blockers or nitroglycerin, which, according to the DeFACTO study, increase CT-FFR specificity^41^.

We are aware that this study has some limitations. First, it was a single-centre study, so comparisons with international, multi-centre studies should be interpreted with caution. Second, although this study was based on a prospective registry, CT-FFR validation was applied retrospectively to those patients who received further ischaemia assessment, which may have led to referral bias. Third, in our study we worked with high-end CT scanners using a 128-slice dual-source, a 192-slice dual-source or a 384-slice dual-source CT scanner, so our results may not be applicable to lower resolution scanners. Finally, the software used to calculate CT-FFR is currently approved for research purposes only and future versions approved for clinical use are likely to have improved features.

## Conclusion

This study undeniably highlights the considerable potential of merging CT-FFR and CCTA, offering a promising non-invasive avenue for accurately detecting ischemia at the coronary artery level. The findings strongly indicate that this combined approach can enhance diagnostic accuracy, thereby significantly influencing clinical decision-making. Nonetheless, it’s essential to recognize that while these results are promising, additional research and technical advancements are warranted to validate and build upon these findings further.

## Data Availability

The datasets analyzed during the current study are not publicly available due to local data protection regulations but are available for further analysis in the setting of cooperation on reasonable request, which can be submitted to the corresponding author.

## Abbreviations

AUC: Area under the ROC
BMI: Body mass index
CAD: Coronary artery disease
CCTA: Coronary computed tomography angiography
CFD: Computational fluid dynamics
CMR: Cardiovascular magnetic resonance
CT-FFR: Computed tomography derived fractional flow reserve
ESC: European society of cardiology
ICA: Invasive coronary angiography
iFFR: Invasively measured fractional flow reserve
NPV: Negative predictive value
MACE: Major adverse cardiac event
ML: Machine learning
PPV: Positive predictive value
PTP: Pre-test probability
ROC: Receiver-operating characteristic
MP-SPECT: Myocardial perfusion single-photon emission computed tomography
